# Predictors of pulmonary hypertension in patients with hypersensitivity pneumonitis

**DOI:** 10.1186/s12890-023-02347-1

**Published:** 2023-02-09

**Authors:** Mohamed Abdelhakim Elnady, Reem Elkorashy, Afnan Nabil, Eman Kamal Ibrahim

**Affiliations:** Faculty of Medicine, Cairo University, Almaadi, Cairo, Egypt

**Keywords:** Hypersensitivity pneumonitis, Pulmonary hypertension, HRCT chest

## Abstract

**Background:**

Hypersensitivity pneumonitis (HP) is an immunologically induced inflammation of the lung parenchyma that occurs in susceptible individuals in response to a variety of antigens. Repeated exposures to the causative antigens lead to chronic HP. The condition could be complicated with pulmonary hypertension (PH).

**Methodology:**

60 patients with established diagnosis of HP were included, clinical examination, high resolution computed tomography (HRCT) of chest, arterial blood gases, six minute walking test (6MWT), desaturation index, spirometry, echocardiography were performed to all patients and right heart catheter was done for patients with high echo probability of PH.

**Results:**

The mean age of patients was 41.67 ± 13.4 years with female predominance 83.3% of patients had history of raising birds. 71.7% of cases suffered from resting hypoxia with oxygen saturation 89 ± 11% with desaturation index 9% ± 8%, Echo probability of PH ranged from low to high 71.67, 21.67 and 6.67% respectively, mean pulmonary artery systolic pressure was 63.65 (18.48) mmHg. PH was diagnosed in 17 (28.33%) patients. HP with PH patients were significantly more symptomatic with predominant fibrotic pattern in HRCT chest *P* < 0.001, 82% of them had hypoxia *P* < 0.001 with significant desaturation after 6MWT *P* = 0.001. Predictors of PH in study group were fibrotic pattern in HRCT chest and hypoxia OR = 62.22, *P* < 0.001; 49.2, *P* < 0.001 respectively.

**Conclusion:**

PH was prevalent in 28.33% of patients with HP, predictors of development of PH were fibrotic pattern in HRCT chest and hypoxia.

*Trial registration*: Retrospectively registered, registration number is NCT05458635, date of registration 07/12/2022.

**Supplementary Information:**

The online version contains supplementary material available at 10.1186/s12890-023-02347-1.

## Background

Hypersensitivity pneumonitis (HP) is a complex immune mediated disorders caused by repeated inhalation of and sensitization to wide range of antigens including organic particles and chemical compounds, leading to exaggerated immuneresponse [1]. Hypersenstivity pneumonitis is a common form of diffuse parenchymal lung diseases in Egypt [2].

HP is classified into two subtypes according to computed tomography findings into fibrotic and non-fibrotic HP. Diagnosis of HP is challenging especially the fibrotic subtype. A multidisciplinary approach including exposure history, HRCT findings, BAL lymphocytosis over 50% and lung tissue biopsy can be used to determine the likelihood of hypersensitivity pneumonitis [3].

The inflammatory process in HP affect small airways and lung parenchyma with development of pulmonary fibrosis, which lead to impairment of exercise capacity and pulmonary hypertension (PH) [4], due to subsequent affection of oxygen diffusion [5].

Pulmonary hypertension has been found as a complication of a number of diseases affecting the lung interstitium, including hypersensitivity pneumonitis, with subsequent affection of the mortality [6]. Rationale of the study is to find predictors of the development of pulmonary hypertension in patients with hypersensitivity pneumonitis.

## Methods

A cross sectional study that was held in a tertiary referral center “Kasr Alainy hospital, Department of pulmonary medicine, ILD unit, approved from research ethical committee, Cairo University (MS-131-2019). It included 60 patients with established diagnosis of hypersensitivity pneumonitis coming for follow up in ILD unit during the period from August 2019 to October 2020.

Diagnosis of HP is based on An official ATS/JRS/ALAT clinical practice guideline, 2020 [3]. Other forms of interstitial lung diseases were excluded. All patients were clinically evaluated regarding WHO functional class, MMRC dyspnea score syncope, palpitation and easy fatigability.

Assessment of oxygenation status by arterial blood gases sampling, hypoxia was considered when PO_2_ in ABGs < 60 mmHg &/or Desaturation < 90% [7].

Six minutes walk test (6MWT) was performed according to ATS 2002 guidelines for all patients as a marker of exercise tolerance with assessment of desaturation difference between the baseline SPO_2_ and post exercise SPO_2_ [8].

Pulmonary function test in the form of spirometry by Master Screen PFT 2012, CareFusion 234 GmbH, Germany (V-781267-057 version 03.00), Restriction was classified as mild, moderate and severe (FVC% predicted 80–60%, 40–60% and < 40% respectively) according to ATS guidelines 1994 [9].

HRCT chest was done using (Siemens 16-channel). HRCT chest findings which are suggestive of HP include; ground-glass. Nodular opacities, air-trapping in the mid to upper portion of the lung lobes, head-cheese sign (combination of GGO and mosaic parenchyma),Peribronchial consolidations, fibrosis, Cystic changes, fibrotic HP includes the following patterns: reticulation, traction bronchiectasis, volume loss, with or without evidence of honeycombing [10, 11]. Echocardiography was done to all patients using Siemens Acuson X300 PE Ultrasound machine, the following parameters were measured; PASP based on the peak tricuspid regurgitation velocity, assessment of the right ventricular size, pressure overload and function, the pattern of blood flow velocity out of the right ventricle, the diameter of the pulmonary artery and an estimate of right atrial pressure, diameter of left ventricle and atrium with assessment of contractile function, valvular thickness and excursion, diameter and collapsibility of I.V.C.

Pulmonary hypertension was diagnosed by echocardiography based on evaluation of the echocardiographic probability of pulmonary hypertension, according to ESC/ERS guidelines, 2015 [12]. Low echocardiogrphic probability of PH is considered when peak tricuspid regurgitation velocity ≤ 2.8 (m/s) or not measurable in absence of other echo PH signs. Intermediate probability is defined as peak tricuspid regurgitation velocity ≤ 2.8 (m/s) or not measurable in presence of other echo PH signs OR when peak tricuspid regurgitation velocity 2.9–3.4 (m/s) in absence of other echo PH signs. High echocardiogrphic probability of PH is considered when peak tricuspid regurgitation velocity 2.9–3.4 (m/s) in association of other echo PH signs OR when peak tricuspid regurgitation velocity > 3.4 (m/s) [12]. Echocardiographic signs suggesting pulmonary hypertension used to assess the probability of pulmonary hypertension in addition to tricuspid regurgitation velocity measurement include; Right ventricle/ left ventricle basal diameter ratio > 1.0, Flattening of the interventricular septum (left ventricular eccentricity index > 1.1 in systole and/or diastole), Right ventricular acceleration time < 105 ms and/or midsystolic notching, Early diastolic pulmonary regurgitation velocity > 2.2 m/s, PA diameter > 25 mm, Inferior cava diameter > 21 mm with decreased inspiratory collapse (< 50% with a sniff or < 20% with quiet inspiration), Right atrial area (end-systole) > 18 cm^2^ [12].

Right heart catheterization was performed for 4 cases with high probability of pulmonary hypertension using multi-lumen swan ganz catheter. PH is defined as an increase in mean pulmonary arterial pressure (mPAP) ≥ 25 mmHg at rest as assessed by right heart catheterization (RHC). Pre-capillary PH, defined by a pulmonary artery wedge pressure (PAWP) ≤ 15 mmHg and a PVR. 3 Wood units (WU) [12].

Patients were classified into two groups based on echocardiography; HP without PH (low probability for PH) included 43 patients and HP with PH including 17 patients (intermediate probability = 13 patients) and (high probability = 4 patients).

### Statistical methods

The data statistically analyzed using Minitab for windows (Minitab Inc., 2013, Pennsylvania, USA), the continues data represented as mean (SD) or median (IQR), while categorical data as number and percentage, independent t-test, or Kruskal Wallis test was used to compare between parametric data, while Chi square test was used with non parametric data. Logistic regression model with and without adjustment for age, sex and bird exposure was used to find the independent predictors for PHT. All tests were two sided, *P* < 0.05 considered significant.

## Results

The study included 60 patients with hypersensitivity pneumonitis. The mean age of patients was 41.67 ± 13.4years, with female predominance (88.3%). Most of the patients live in rural areas 85%, 83.3% of patients had history of significant exposure to organic materials, “Birds’ droplets” whether poultry or pigeons. Only 10 cases of 60 had no overt exposures and were considered cryptic. Regarding the patients’ symptoms, all patients were complaining of dyspnea and cough. The mean duration of complain is 3 years. According to the WHO functional class, the percentage of patients were 3.3%, 63.3 %, 28.3%, 5% respectively for function class 1, 2, 3, and 4. Spirometric assessment was performed and revealed restrictive pattern in 91.67% of cases (n = 55), while only 5 cases showed normal spirometric pattern, Regarding oxygenation, 71.7% (n = 43) of cases suffered from resting hypoxia with mean oxygen saturation 89 ± 11%, and post exercise the saturation declined to mean 83 ± 12%, with desaturation index about 9±8%.Exercise capacity was evaluated by mean of 6 minute walking test, and showed mean distance 261.35 ± 68.1 m. The probability of Echo in detection of PH ranged from low to high with frequency about 71.67, 21.67 and 6.67 % respectively. In those with pulmonary hypertension, the mean (SD) PASP was 63.65 (18.48) mmHg (Table 1)

**Table 1:** Characters of patients with chronic hypersensitivity pneumonia

Factors	Total (n = 60)	
	N	%
Clinical features		
Syncope	9	15
Palpitation	24	40
Easy fatigability	35	58.33
Clubbing	21	35
Crepitation on auscultation	41	68.33
HRCT pattern		
Typical non-fibrotic HP	46	76.66
Typical fibrotic HP	14	23.34
mPASP	63.65	18.48
Echo probability		
High	4	6.67
Intermediate	13	21.67
Low	43	71.67

ABG	Mean	SD
PO_2_	68.97	15.20
PCO_2_	40.33	5.63
SO_2_%	89	11
Aa PO_2_ gradient	51.85	46.42

Continues data represent as mean and stander deviation (SD), and categorical data as number and percentage

*N* Number

PH was diagnosed in 17 patients (28.33%).Based on echocardiographic probability of PH according to ESC/ERS guidelines, 2015 [12]. 13 patients with intermediate and 4 patients with high probability of PH. Right heart catheter was done to 4 patients with high echo propability for PH. Measured mPAP (73, 69, 70, 68), pulmonary artery wedge pressure was (6, 11, 9, 12) respectively (Table 2). The 4 cases with pulmonary hypertension were of precapillary type and their HRCT pattern was the fibrotic.

**Table 2:** RHC data of cases with high echocardiographic probability of PH

	RAP	RVP	mPAP	PAWP	CO	CI	SV	PVR	SVO_2_ (%)
CASE 1	13	55	73	6	3.2	2	35.5	20	48
CASE 2	9	49	69	11	4.3	2.7	50	13.4	72
CASE 3	16	50	70	9	4.2	2.2	42	14.5	55
CASE 4	21	44	68	12	4.8	2.8	50	11.6	63

*RAP* Right atrial pressure, *RVP* Right ventricular pressure, *mPAP* Mean pulmonary artery pressure, *PAWP* Pulmonary artery wedge pressure, *CO* Cardiac output, *CI* Cardiac index, *SV* Stroke volume, *PVR* Pulmonary vascular resistance, *SVO*_*2*_ Venous oxygen saturation

**Fig. 1:**
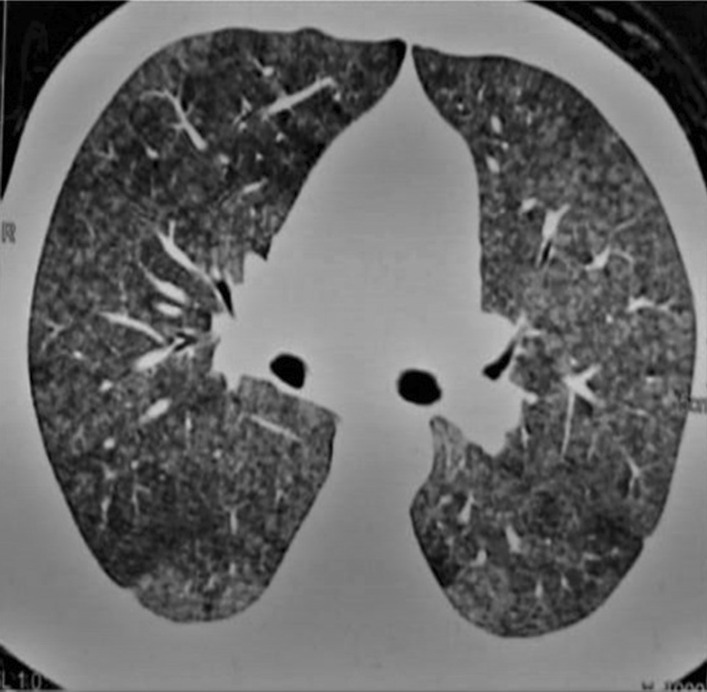
Female patient 36 years with nodular pattern in HRCT chest, low probability of PH in echocardiography and mild restriction spirometry

**Fig. 2:**
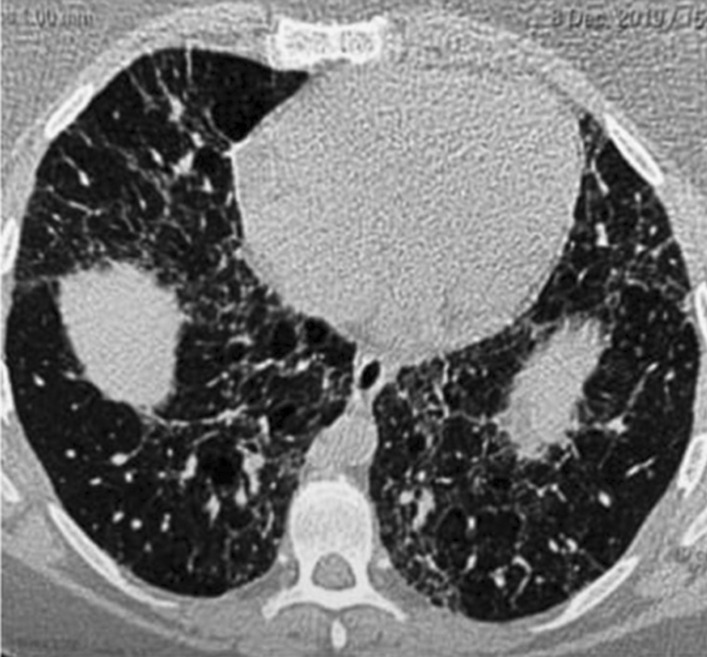
Female patients 41 years with fibrotic HP, intermediate probability of PH in echocardiography and severe restriction

Patients with HP and PH were significantly characterized by high grade of dyspnea, syncope, palpitation and easy fatigability *P* = 0.003, 0.001, 0.002 and 0.01 respectively. Moreover, HRCT chest of them significantly showed fibrotic pattern more than mosaic or nodular pattern (Figs. 1, 2) *P* < 0.001.However the physical performance of patients with PH was lower than the other group (mean (SD) of 6MWTD was 231 (75) versus 272 (63) meter) but difference did not reach the significant level *P* = 0.08. More than 2/3 of them had hypoxia (less than 90%) in comparison with other group *P* < 0.001, as well as significant decrease of oxygen saturation before and after 6MWT *P* = 0.001 for both. In the same line ABGs finding suggested that patients with PH had significant hypoxemia and hypocapnea than the other group *P* < 0.01 for all. (Table 3).

**Table 3:** Demographic data, clinical, radiological and functional characteristics of HP patients with and without PH

Factors	HP without PH (n = 43)		HP with PH (n = 17)		*P*
	Mean	SD	Mean	SD	
Age	42.6	13.9	39.3	12	0.36^$^
Sex	N	%	N	%	
Male	6	13.95	1	5.88	0.38^#^
Female	37	86.05	16	94.12	
Bird exposure	N	%	N	%	
Yes	38	88.37	12	70.59	0.11^#^
	Median	IQR	Median	IQR	
Dyspnea score (MMRC)	2	(2–2)	3	(2–3)	**0.003**^§^
Duration (years)	2	(1–3)	3	(2–4)	0.27^§^
Clinical features	N	%	N	%	
Syncope	2	4.65	7	41.18	**0.001**^#^
Palpitation	12	27.91	12	70.59	**0.002**^#^
Easy fatigability	21	48.84	14	82.35	**0.01**^#^
Clubbing	12	27.91	9	52.94	0.06^#^
Crepitation on auscultation	27	62.79	14	82.35	0.12^#^
HRCT pattern	N	%	N	%	
Typical non-fibrotic HP	41	95.35	5	29.41	** < 0.001**^#^
Typical fibrotic HP	2	4.65	12	70.59	
O_2_ saturation %	N	%	N	%	
Less than 90%	3	6.98	14	82.35	** < 0.001**^#^
More than 90%	40	93.02	3	17.65	
FVC%	**60%**	**17%**	**53%**	**16%**	**0.18**^$^
FVC restriction degree*	1	(1–2)	2	(1–2)	0.54^§^
FEV1%	0.59	0.17	0.60	0.16	0.96^$^
SO_2_ in RA	0.93	0.08	0.81	0.12	**0.001**^$^
Min SO_2_ in 6MWT	0.87	0.09	0.71	0.13	**0.001**^$^
Desaturation index	0.04	(0.03–0.1)	0.11	(0.07–0.19)	**0.001**^§^
6MWD	271.90	63.20	231.10	74.70	0.08^$^
PO_2_	75.30	11.70	52.80	10.40	** < 0.001**^$^
PCO_2_	41.63	4.54	37.06	6.86	**0.01**^$^
FIO_2_	0.22	0.03	0.28	0.05	** < 0.001**^$^
Aa PO_2_ gradient	23.73	(15.1–31.9)	106.24	(91.6–119.2)	** < 0.001**^§^

Bold value indicates FVC %

*FVC restriction degree 1 = normal and mild restriction, 2 = moderate and sever restriction

*P* < 0.05 considered significant

^$^Independent t-test

^§^Kruskal Wallis test

^#^Chi square test

The likelihood of PH increased 62 and 49 time more in HP patients with hypoxia (less than 90%) and fibrotic pattern in CT than those without: OR = 62.22, *P* < 0.001; 49.2, *P* < 0.001 respectively. However, after adjustment for age, sex and bird exposure, the likelihood of PH still higher in presence of hypoxia and fibrosis in CT, OR = 28 and 17, *P* = 0.01 and 0.02 respectively. (Table 4).

**Table 4:** Predictors of pulmonary hypertension in patients with HP

Factors	OR	95% CI	*P*
A—Unadjusted model^$^			
O_2_ saturation < 90%	62.22	(11.2299, 344.7583)	< 0.001
Fibrotic pattern in CT	49.20	(8.4520, 286.3975)	< 0.001
B—Adjusted model^§^			
Typical fibrotic pattern in HRCT	17.03	(1.5524, 186.7917)	0.02
O_2_ saturation < 90%	28.07	(2.1895, 359.8159)	0.01

*CI* Confidence interval, *OR* Odd ratio

*P* considered significant if < 0.05

^$^Goodness of fit test: Pearson test, X^2^ = 60, *P* = 0.4

^§^Goodness of fit test: Hosmer Lemeshow test, X^2^ = 8.5, *P* = 0.38

## Discussion

The current study included 60 patients with hypersensitivity pneumonitis to assess the predictors and factors associated with pulmonary hypertension in patients with HP.

The descriptive data showed that the mean age of patients was 41.67 years which is considered middle age, with a female predominance (88.33%), mostly resident in rural areas (85%), history of raising birds found in (83.33%) and (16.67%) of patients did not show history of exposure to a known causative agent, so it was considered as cryptic HP. These results are matched with the study conducted by Akl et al. in Egypt, in which females were found to be affected ten times more than males with a ratio 10:1, and the mean age of affection was 42.72 years ± 12.54 and most of patients had history of raising birds (78.12%) [13].

Others found no significant sex differences in HP patients, with the mean age was 61.9 years which was older than our patients, with 64% of patients were bird breeders [14].

This may reflect the characteristics of the exposed working population burden [15], where the variability in the clinical presentation and natural history is dependent on the causative agent [16]. In Egypt, it is known that ladies in Egyptian rural areas usually breed birds to increase their financial resources which may explain the female predominance in the current study. The repeated and prolonged inhalation of different types of substances especially organic dust until becoming sensitized and hyper-responsive to the antigen may take long duration [17], thus the disease appears in the middle age. However the diversity of antigens that can cause hypersensitivity pneumonitis which may reach more than 200 known causative agent may explain that there may be exposures to antigens that are still not discovered [18].

As regards the clinical presentation, all the patients were complaining of cough and dyspnea with different grades (90% of them were between mild and moderate grades) this is consistent with the study conducted by Lima et al. [19] in which the main clinical features were dyspnea in 85%, and dry cough in 78%. Also in a considerable percentage of patients, there were easy fatigability and clubbing as also found in another study [20].

A smaller percentage of patients (15%), experienced syncope which is uncommon symptom in hypersensitivity pneumonitis, but it may be related to development of pulmonary hypertension. It is used as one of the markers of severity to assess mortality in the first year of diagnosis in PH [12]. It denotes progressive decline in the right ventricular function [21].

HRCT scan findings of the current study population revealed the following patterns; ground glass opacities GGO (100%), Centri-lobular nodules (nodular pattern) (38.33%), Air trapping (mosaic pattern) (38.33%), Fibrotic pattern (23.33%). Similar results were found in El-Kareem et al. study in which the most predominant patterns were isolated GGO or in combination with either nodules or air trapping [2]. This was also matched with results of another study [22].

The mean arterial oxygen saturation in the study group was 89 ± 11% on resting position and there were exercise desaturation reaching a mean of 83 ± 12%, finding that was consistent with Lima et al. study [19].

This exercise desaturation is explained by the increase in the oxygen demand [23] and concomitant decrease in the transient time leading to decrease oxygen uptake [24].

The most accessible, most widely used non-invasive tool to screen for pulmonary hypertension (PH) in patients with lung diseases is echocardiography [25]. Patients with echocardiographic high probability of PH, are candidates to right heart catheterization (RHC), especially if treatment plan would be affected by RHC results [25]. However most patients with ILD would not benefit from RHC at the time of diagnosis, and so echocardiography is considered the technique of choice for initial PH evaluation [12, 25].

In the current study PH was considered in 17 patients (28.33%) based on echocardiographic signs and probability of PH (13 patients with intermediate and 4 patients with high probability of PH).

Patients were classified into two groups HP with and without PH (based on echocardiographic probability for PH), comparison of both groups revealed that patients with PH showed significant increase in their symptoms mainly dyspnea, syncope, palpitation and easy fatigability. With predominant fibrotic pattern in HRCT chest than other patterns, non significant decrease of 6MWTD, about 82% of them had hypoxia (O_2_ saturation < 90%) in comparison with other group, as well as significant reduction of oxygen saturation during and after 6MWT. Significant hypoxemia and hypocapnea in ABGs than the other group.

The first research concerned with PH in HP patients was conducted by Koschel et al. it revealed that 19% of patients with fibrotic HP had PH based on estimated PASP > 50 mmHg by echocardiography, This was matched to our results [26].

Another study evaluated the hemodynamics of PH in HP patients by RHC documented PH in 50% of patients with fibrotic HP (mPAP ≥ 25 mmHg) and sever PH (mPAP > 35 mmHg) in only 10%. Patient with PH were significantly hypoxic with reduction of FVC and DLCO and significant desaturation after 6MWD [27].

Walscher et al. found PH in 9.5% of HP patients, but they depend on medical reports only to define PH which was not sufficient [28].

More recent study conducted on 70 patient with HP considered PH diagnosis in 37% of patients based on echocardiography, authors noticed that patient with HP and PH has significant reduction of resting PO_2_ and 6MWD and O_2_ desaturation rate, this was close to our results [4].

The discrepancy of data between studies concerning PH in HP could be explained by differences in patients ҆ age, percentage of patients fibrotic HP and time from disease onset to diagnosis.

Regarding predictors of PH, our results revealed that the likelihood of PH increased 62 and 49 time more in HP patients with hypoxia (less than 90%) and fibrotic pattern in CT,even after adjustment for age, sex and bird exposure, the likelihood of PHT still higher in presence of hypoxia and fibrosis in CT, OR = 28 and 17.

The development of pulmonary hypertension in chronic lung diseases is multifactorial, the main factor is hypoxic vasoconstriction of pulmonary vessels to shift blood to well ventilated areas to optimize ventilation-perfusion matching, sustained hypoxia promotes intracellular mediators and hypoxia inducible factor 1α (HIF-1α) leading to increase vascular remodeling and increase pulmonary vascular resistance with further increase in pulmonary artery pressure [29].

Dybowska et al. found that fibrotic HP was not a predictor of PH on echocardiogrsphy when they use PASP 36 mmHg as cut off of diagnosis of PH, but PASP > 50 mmHg was exclusively observed in fibrotic HP. So, they declared that role of pulmonary fibrosis as predictor of PH in HP was not clear, which is not in line with our findings [4].

This study had several limitations. First, it is a single centre study with potential selection bias of the patients. Second, small number of the study group and that we depend on the echocardiography signs and probability for diagnosis of pulmonary hypertension and that RHC was performed in 4 cases (high echo probability of PH). But, based on ESC/ERS guidelines, 2015 Potential indications for RHC in advanced lung disease are (1) To confirm diagnosis or exclude PH in candidates for surgical treatments (transplantation, lung volume reduction), (2) If PAH or CTEPH were suspected, (3) RV failure and (4) inaccurate echocardiographic findings in highly suspicious cases with potential therapeutic implications [12]. So, we reserve RHC for patients with high echocardiographic probability of PH while patients with intermediate echocardiographic probability for PH will be followed closely if there is any clinical deterioration we will proceed to RHC.

## Conclusions

PH was found in 28.33% of patients with HP based on echocardiographic probability of PH. Predictors of development of PH in our study group were hypoxia and fibrotic pattern in HRCT chest.

## Supplementary Information

**Additional file 1.** The main results of patients; age, sex, clinical features, HRCT- chest patterns, pulmonary functions, echocardiographic findings and probability of PH, 6MWD and degree of desaturation.

## Data Availability

Data of the current study are included in the published article and is attached as Additional file 1.

## Abbreviations

HP: Hypersensitivity pneumonitis
PH: Pulmonary hypertension
HRCT chest: High resolution computed tomography of chest
ABGs: Arterial blood gases
6MWT: 6 Minute walking test
PASP: Pulmonary artery systolic pressure
ILD: Interstitial lung disease
MMRC: Modified medical research council
WHO: World Health Organization
PO_2_: Partial pressure of oxygen
ATS: American Thoracic Society
SPO_2_: Oxygen saturation
PFT: Pulmonary function test
GGO: Ground glass opacification
IVC: Inferior vena cava
mPAP: Mean pulmonary artery pressure
RHC: Right heart catheter
6MWD: 6 Minute walking distance
FVC: Forced vital capacity
DLco: Diffusing lung capacity for carbon monoxide
RAP: Right atrial pressure
RVP: Right ventricular pressure
PAWP: Pulmonary artery wedge pressure
CO: Cardiac output
CI: Cardiac index
SV: Stroke volume
PVR: Pulmonary vascular resistance
SVO_2_: Venous oxygen saturation
