# Supplementary material for: Predictors of pulmonary hypertension in patients with hypersensitivity pneumonitis
Source: BMC Pulm Med. 2023 Feb 9;23:61. doi: 10.1186/s12890-023-02347-1 (PMC9909854; doi:10.1186/s12890-023-02347-1)
Supplement: Supplementary file 1 — Additional file 1. The main results of patients; age, sex, clinical features, HRCT- chest patterns, pulmonary functions, echocardiographic findings and probability of PH, 6MWD and degree of desaturation. [file 12890_2023_2347_MOESM1_ESM.pdf]

patient ID	age	sex	residence	history of birds exposure	symptoms and duration	dyspnea score	syncope	palpitation	easy fatigability	so2 on room air	clubbing	crepitation on auscultation	accentuated S2	HRCT pattern	FVC %	FEV1 %	FEV1/FVC
1	42	female	Giza	yes (pigons)	cough , dyspnea 3 years	2	no	yes	yes	89%	yes	yes	yes	fibrotic HP	59%	70%	62%
2	49	female	Shubra Elkhama	yes (poultry)	cough , dyspnea 4 years	4	yes	yes	yes	80%	yes	yes	yes	fibrotic HP	47%	55%	68%
3	59	female	Almaadi	yes (pigons)	dyspnea2 years	2	no	no	no	92%	no	no	no	nodular HP	63%	77%	71%
4	15	female	Aswan	no	cough , dyspnea 1 year	3	yes	yes	yes	85%	no	yes	yes	nodular HP	71%	71%	81%
5	38	female	Elwarraq/Giza	yes (pigons)	cough , dyspnea 3 years	2	no	no	yes	98%	no	no	no	nodular HP	49%	50%	72%
6	55	female	Giza	yes (poultry)	cough , dyspnea 3 years	2	no	no	yes	96%	no	no	no	nodular HP	67%	74%	75%
7	57	female	Giza	yes (poultry)	cough , dyspnea 5 years	2	no	no	yes	97%	no	yes	no	nodular HP	64%	72%	70%
8	30	female	Giza	no	dyspnea 3 years	2	no	no	no	95%	no	no	yes	nodular HP	64%	66%	76%
9	21	female	Giza	yes (pigons)	cough , dyspnea 3 years	3	yes	yes	yes	80%	yes	yes	yes	fibrotic HP	37%	55%	58%
10	50	female	Giza	yes (poultry)	cough , dyspnea 2 years	1	no	no	no	94%	no	no	no	mosiac HP	78%	73%	80%
11	34	female	Nagaa Hamady	yes (poultry)	cough , dyspnea 3 years	3	yes	yes	yes	80%	no	yes	yes	nodular HP	60%	62%	70%
12	56	female	Giza	yes (poultry)	cough , dyspnea 2 years	2	no	no	no	95%	no	no	no	mosiac HP	73%	79%	77%
13	47	male	Embaba	no	cough , dyspnea 1 years	3	no	no	no	94%	no	no	no	mosiac HP	52%	56%	86%
14	40	female	Fayoum	yes (pigons)	cough , dyspnea 2 years	2	no	no	no	94%	no	no	yes	nodular HP	77%	88%	81%
15	51	female	Giza	yes (pigons)	cough , dyspnea 4 years	3	no	yes	yes	83%	no	yes	yes	fibrotic HP	50%	86%	81%
16	53	female	Bany Swaf	yes (poultry)	cough , dyspnea 2 years	2	no	no	yes	92%	no	yes	no	mosiac HP	56%	64%	67%
17	54	female	Embaba	yes (pigons)	cough , dyspnea 2 years	2	yes	no	no	93%	no	no	no	mosiac HP	69%	62%	71%
18	57	female	doki	yes (pigons)	cough , dyspnea 2 years	1	no	no	yes	95%	no	no	no	mosiac HP	77%	69%	70%
19	37	female	Giza	yes (poultry)	cough , dyspnea 1 year	2	no	no	yes	93%	no	no	no	nodular HP	63%	67%	72%
20	64	female	Giza	yes (poultry)	cough , dyspnea 3 years	2	no	no	no	85%	no	yes	yes	mosiac HP	68%	63%	66%
21	61	female	Giza	yes (poultry)	cough , dyspnea 2 years	2	no	no	yes	85%	no	yes	yes	fibrotic HP	45%	31%	66%
22	53	female	Embaba	yes (poultry)	cough , dyspnea 2 years	3	no	yes	yes	80%	yes	yes	yes	fibrotic HP	31%	45%	55%
23	13	male	elsharkia	yes (poultry)	cough , dyspnea 1 years	2	no	yes	yes	94%	no	no	no	mosiac HP	41%	45%	48%
24	40	female	helwan	yes (poultry)	cough , dyspnea 2 years	2	no	no	no	94%	no	no	no	mosiac HP	58%	63%	77%
25	32	female	Giza	yes (poultry)	cough , dyspnea 3 years	2	no	no	no	96%	yes	yes	no	mosiac HP	71%	67%	88%
26	35	female	Giza	yes (poultry)	cough , dyspnea 2 years	3	no	no	yes	97%	no	yes	no	nodular HP	42%	42%	86%
27	50	female	Giza	yes (poultry)	cough , dyspnea 2 years	2	no	no	no	96%	no	yes	no	nodular HP	65%	77%	99%
28	60	female	Giza	yes (poultry)	cough , dyspnea 20 years	2	no	no	no	92%	yes	yes	no	nodular HP	81%	74%	77%
29	34	female	helwan	yes (poultry)	cough , dyspnea 2 years	2	no	no	no	99%	no	no	no	fibrotic HP	28%	28%	97%
30	30	male	qina	yes (pigons)	cough , dyspnea 1 years	2	no	no	no	90%	no	yes	no	mosiac HP	46%	49%	80%
31	33	female	elmenia	yes (poultry)	cough , dyspnea 2 years	2	no	no	no	96%	no	no	no	mosiac HP	72%	65%	91%
32	50	female	Embaba	no	cough , dyspnea 2 years	2	no	no	no	97%	no	yes	no	nodular HP	49%	46%	80%
33	53	female	cairo (Qanater)	yes (poultry)	cough , dyspnea 4 years	2	no	no	no	94%	no	no	no	nodular HP	91%	89%	82%
34	54	female	Giza	yes (poultry)	cough , dyspnea 2 years	2	no	no	no	93%	no	no	no	mosiac HP	51%	55%	90%
35	25	female	Giza	yes (poultry)	cough , dyspnea 1 years	2	no	no	no	98%	yes	yes	no	nodular HP	37%	34%	77%
36	47	female	Giza	no	cough , dyspnea 2 years	3	yes	yes	yes	61%	yes	yes	yes	fibrotic HP	*	*	*
37	37	female	Giza	no	cough , dyspnea 8 years	4	yes	yes	yes	67%	yes	yes	yes	fibrotic HP	*	*	*
38	60	male	cairo (Qanater)	no	cough , dyspnea 2 years	2	no	no	no	88%	yes	yes	no	mosiac HP	71%	65%	82%
39	45	female	elmenia	yes (poultry)	cough , dyspnea 2 years	2	no	no	no	98%	no	yes	no	nodular HP	45%	52%	99%
40	35	female	Giza	yes (poultry)	cough , dyspnea 15 years	4	yes	yes	yes	50%	yes	yes	yes	fibrotic HP	20%	29%	55%
41	42	female	Giza	no	cough , dyspnea 6 months	2	no	no	no	97%	no	no	no	nodular HP	54%	39%	89%
42	63	female	Giza	yes (poultry)	cough , dyspnea 3 years	2	no	no	no	90%	no	yes	no	mosiac HP	70%	79%	94%
43	14	male	Embaba	yes (pigons)	cough , dyspnea 1 years	2	no	yes	no	97%	yes	yes	no	nodular HP	49%	41%	81%
44	25	female	cairo (shoubra)	yes (pigons)	cough , dyspnea 4 years	3	no	yes	yes	50%	yes	yes	no	mosiac HP	29%	30%	92%
45	29	male	cairo (el maadi)	no	cough , dyspnea 4 months	2	no	yes	yes	86%	yes	yes	no	fibrotic HP	66%	67%	68%
46	32	female	Bany Swaf	yes (poultry)	cough , dyspnea 4 years	3	no	no	yes	94%	no	yes	no	mosiac HP	100%	90%	84%
47	54	female	Elwarraq/Giza	yes (pigons)	cough , dyspnea 4 years	3	yes	yes	yes	81%	yes	yes	yes	fibrotic HP	46%	47%	89%
48	19	male	helwan	no	cough , dyspnea 1 year	2	no	yes	yes	98%	yes	yes	no	nodular HP	63%	74%	99%
49	36	female	Giza	yes (poultry)	cough , dyspnea 4 years	3	no	no	yes	98%	yes	yes	no	nodular HP	28%	32%	98%
50	49	female	Embaba	yes (poultry)	cough , dyspnea 2 years	3	no	no	yes	92%	yes	yes	no	nodular HP	55%	49%	85%
51	31	female	Embaba	yes (poultry)	cough , dyspnea 6 years	3	no	yes	yes	85%	yes	yes	no	mosiac HP	47%	48%	61%
52	35	female	Elfayoum	yes (pigons)	cough , dyspnea 6 years	3	no	yes	yes	91%	yes	yes	no	mosiac HP	40%	41%	90%
53	40	female	Elfayoum	yes (poultry)	cough , dyspnea 1 year	2	no	no	yes	70%	yes	yes	yes	fibrotic HP	43%	49%	83%
54	38	female	Giza	yes (poultry)	cough , dyspnea 1 year	2	no	no	yes	96%	no	no	no	mosiac HP	69%	68%	83%
55	52	female	Giza	yes (poultry)	cough , dyspnea 1 year	2	no	yes	yes	95%	no	yes	no	mosiac HP	66%	63%	77%
56	42	female	Giza	yes (poultry)	cough , dyspnea 1 year	2	no	yes	yes	95%	no	yes	no	nodular HP	60%	41%	59%
57	50	female	Giza	yes (poultry)	cough , dyspnea 1 year	2	no	yes	no	97%	no	yes	no	mosiac HP	59%	56%	84%
58	26	female	Giza	yes (pigons)	cough , dyspnea 2 year	2	no	yes	yes	95%	no	yes	no	mosiac HP	67%	69%	94%
59		female	Giza	yes (poultry)	cough , dyspnea 1 year	3	no	yes	yes	88%	no	yes	yes	fibrotic HP	74%	81%	90%
60	16	female	Giza	yes (poultry)	cough , dyspnea 1 year	3	no	yes	no	95%	no	yes	no	mosiac HP	90%	89%	82%

minimum SO2 in 6MWT	Distance in 6MWD	Indirect signs of PHTN	echo propapility of PHTN	PO2 in ABG	mPAP by RHC IF indicated	lymphocytes% in BAL If done	desaturation degree
82%	250 m	PTRV =3m/S , dilated RV	HIGH	55			7%
61%	110 m	IVC Diameter 3cm , dilated RV , PAsP = 70	HIGH	48	Patient died		19%
82%	270 m	no	low	85			10%
77%	280 m	PTRV =NOT measurable , PAsP = 60	Intermediate	55	52 mmHg	60%	8%
97%	300 m	no	low	77			1%
94%	350 m	no	low	80			2%
97%	330 m	no	low	81			0%
84%	310 m	PTRV =NOT measurable , PAsP = 45 , IVC = 24mm	Intermediate	77			11%
50%	125 m	PTRV =NOT measurable , PAsP = 65 , IVC =30mm	Intermediate	54			30%
90%	270 m	no	low	72			4%
53%	300 m	PTRV =NOT measurable , PAsP = 49 , dilated RV	Intermediate	55			27%
94%	270 m	no	low	78			1%
91%	330 m	no	low	87			3%
91%	290 m	no	low	80			3%
79%	200 m	PTRV =NOT measurable , PAsP = 80 , dilated RV , RA	Intermediate	58			4%
89%	250 m	no	low	74			3%
94%	300 m	no	low	85			0%
92%	240 m	no	low	83		55%	3%
89%	210 m	no	low	75			4%
80%	230 m	no	low	50			5%
70%	220 m	PTRV =NOT measurable, PAsP = 60,dilated RV, IVC=2.2cm	Intermediate	48			15%
60%	160 m	PTRV =NOT measurable, PAsP = 52,dilated RV,mild pericardia	Intermediate	48			20%
91%	480 m	no	low	85			3%
83%	220 m	yes	Intermediate	55			11%
66%	280 m	no	low	72			30%
94%	270 m	no	low	88			3%
85%	260 m	no	low	71			11%
90%	270 m	no	low	81		lymphocytes 75%	2%
98%	128 m	no	low	88%			1%
61%	220 m	no	low	68			29%
85%	360 m	yes	Intermediate	76	58		11%
93%	300m	no	low	77			4%
91%	280m%	no	low	72			3%
91%	190 m	no	low	68			1%
88%	285m	no	low	71			10%
*	*	yes	HIGH	44			*
*	*	yes	HIGH	41			*
77%	210 m	no	low	71			11%
98%	280 m	no	low	109			0%
*	*	yes	Intermediate	43			*
93%	300 m	no	low	73			4%
85%	180 m	no	low	78			5%
85%	195 m	no	low	82			12%
*	*	no	low	44			*
80%	220 m	yes	Intermediate	52			6%
91%	375 m	no	low	88			3%
60%	130 m	yes	low	44			19%
73%	270 m	no	low	81			25%
81%	270 m	no	low	69			17%
77%	280 m	no	low	70			14%
79%	310 m	no	low	63			6%
87%	270 m	no	low	83			4%
55%	170 m	yes	Intermediate	48			15%
89%	350 m	no	low	75			7%
90%	310 m	no	low	67			5%
91%	290 m	no	low	71			4%
93%	300 m	no	low	69			4%
91%	280 m	no	low	88			4%
80%	310 m	yes	moderate	41			8%
93%	300%	yes	low	50			*
